# Valorization of grape (*Vitis vinifera*) leaves for bioactive compounds: novel green extraction technologies and food-pharma applications

**DOI:** 10.3389/fchem.2023.1290619

**Published:** 2023-12-12

**Authors:** Jyoti Singh, Prasad Rasane, Rajdeep Kaur, Harmandeep Kaur, Ritika Garg, Sawinder Kaur, Sezai Ercisli, Ravish Choudhary, Sona Skrovankova, Jiri Mlcek

**Affiliations:** ^1^ Department of Food Technology and Nutrition, School of Agriculture, Lovely Professional University, Phagwara, Punjab, India; ^2^ Department of Horticulture, Faculty of Agriculture, Ataturk University, Erzurum, Türkiye; ^3^ HGF Agro, ATA Teknokent, Erzurum, Türkiye; ^4^ Seed Science and Technology, ICAR-Indian Agricultural Research Institute, New Delhi, India; ^5^ Department of Food Analysis and Chemistry, Faculty of Technology, Tomas Bata University in Zlín, Zlín, Czechia

**Keywords:** grape leaves, antioxidant, extraction, polyphenols, health, application

## Abstract

Grape leaves, scientifically known as *Vitis vinifera,* the primary by-product obtained after the processing of grapes, are gathered in enormous amounts and disposed of as agricultural waste. For more sustainable agriculture and better food systems, it is crucial to investigate these byproducts’ nutritional values. The primary bioactive compounds present in grape leaves are quercetin, resveratrol, caffeic acid, kaempferol, and gallic acid, which favour pharmacological effects on human health such as antioxidant, anti-inflammatory, anti-obesity, anti-diabetic, and hepatoprotective. Furthermore, grape leaves extract has been used as a functional ingredient for creating both food and non-food products. The aim of the current review is to review the nutritional and phytochemical composition of various varieties of grape leaves, their health-promoting characteristics and their applications. The study also highlights the various extraction techniques including conventional and non-conventional methods for extracting the various bioactive compounds present in grape leaves. Grape leaves bioactives can be extracted using environmentally safe and sustainable processes, which are in line with the rising demand for eco-friendly and healthful products worldwide. These methods are perfectly suited to the changing needs of both customers and industries since they lessen environmental effect, enhance product quality, and offer financial advantages.

## 1 Introduction

Grapes are one of the world’s most valued horticultural crops. As per the information given by the Food and Agriculture Organization of the United Nations (FAO) statistical database, grape production in 2019 was 77 million tons (Li et al., 2022). The production of grapes often known as viticulture is one of the biggest agricultural product. Around 10,000 varieties of grapes can be found worldwide (Ins et al., 2021). China produces the most grapes in the world (Li et al., 2022). Moreover, other countries which focus on the production part of fresh grape leaves are India, Mexico, Iran, Egypt, Brazil and Turkey (Ins et al., 2021). Grape leaves, a vegetative portion of grapes are still neglected by-product of the sector where bioactive components of grapevine are investigated at the grape level (Maia et al., 2019).

Grape (*Vitis vinifera* L*.)* is one of the most significant perennial crops worldwide (Gülcü et al., 2020). Vine leaves have long been consumed both fresh and canned. Vine leaves that have been brined are a good source of organic acids, sugars, and phenolic compounds. The primary output of the vine industry is wine and alcoholic beverages. However, they do not stand alone in the market. *Vitis vinifera* employed in the grape business provides fresh grapes, raisins, juices, jellies, molasses, jam, and dishes prepared with vine leaves. Turkish, Balkan and Middle Eastern cuisines have long been known for their framed dishes made with freshly harvested, stuffed grape leaves (dolma) (Banjanin et al., 2021). There are currently over 2000 distinct cultivars of *Vitis vinifera* grown all over the world and utilised for grape production. About 50% of the grapes grown world-wide are used to make wine, which is a highly strategic industry for the economics of many different nations. The remaining 50% are either dried or used to make grape juice or must (Maia et al., 2019). According to the Food and Agriculture Organization (FAO), approximately 78 million of grapes are produced globally (Madadian et al., 2022).

When grape vines are produced, grape leaves are discarded, which decreases the economic growth of farmers. However, if grape leaves were used for their therapeutic properties, farmers might see a rise in their financial situation as well as environmental benefits for sustainable viticulture (dos Santos Lacerda et al., 2014). In Southwestern Europe, where grape leaves are a major environmental issue since they are released as waste after winemaking. In order to address this issue, bioactive components from Vitis vinifera leaves are extracted and employed (Lantzouraki et al., 2020). Vitamins, anthocyanins, flavonoids, polyphenols, and stillbenoids are some of the secondary bioactive metabolites found in grapes that are used to make supplements and cosmetics (Maia et al., 2019). Numerous researches on the V. vinifera L. plant materials demonstrate their value as a top source of minerals, organic acids (such as malic, oxalic, fumaric, ascorbic, citric, and tartaric acid), polyphenols, enzymes, and other nutrients essential for human nutrition (Pantelić et al., 2017).

Furthermore, oxidative stress caused by both intrinsic factors (e.g., DNA damage) and extrinsic factors such as lifestyle, cigarette smoking, pollution, and radiation, leads to the production of Reactive Oxygen Species (ROS), which in turn results in aging and the development of neurodegenerative disorders, including Parkinson’s Disease and Alzheimer’s Disease. Notably, the polyphenols found in *Vitis vinifera* leaves have the ability to protect against oxidative stress. Activity of endogenous antioxidants are enhanced by polyphenols; other mechanism of polyphenols is by preventing the production of Reactive Oxygen Species (ROS) and can delay or block oxidative stress. Several polyphenols from *Vitis Vinifera* leaves were purified and isolated, this offers the food and pharmaceuticals industries new and profitable prospects (Dresch et al., 2018; Uddin et al., 2020). Generation of free radicals and oxidation are prevented by grape leaf extract which possesses antioxidant activity due to high polyphenolic contents in it. According to the several studies, *Vitis vinifera* leaf extract can reduce the release of IL-8 which is induced in the TNF-α pathway which is a cytokine which trigger inflammation process (Vinceković et al., 2019; Jang et al., 2021). Grapes leaves and grapes are considered a vey rich source of phenolic compounds and have a huge potential for the development of new foods and beverage (Vilela and Pinto, 2019).

## 2 Morphology

The leaf of *Vitis vinifera* is a typical mesomorphic leaf with a dorsoventrally flattened lamina, where it is simple to differentiate between the colouration of the upper surface (green-dark) and lower surface (clear green). Despite some differences in the morphological and anatomical characteristics of *Vitis vinifera* cultivars, this species’ leaves are typically big, alternating, petiolate, and palmately lobed, giving them the appearance of being in the shape of a hand (Cosme et al., 2017). Grapevine leaf tissue includes cuticle, upper andmesophyll, and lower epidermis, which contains spongy and palisade parenchyma with embedded xylem and phloem. The tissue between the two epidermal layers of a leaf is known as the mesophyll. The mesophyll is composed of four to six layers of spongy parenchyma, also known as spongy mesophyll, and a single layer of elongated cells known as palisade parenchyma (Keller, 2020). The ability of the epidermis to protect the leaf from uncontrolled water loss, environmental degradation, and microbial attack is formed by the cuticle, which is a separate layer formed by the incrustation of the outer wall with cutin (Cosme et al., 2017).

Mesophyll, the primary component of a leaf, is a plant’s photosynthetic system. Although it does not have phenolic chemicals, the tissue is primarily chlorophyll rich. The cuticle, a very thin but significant layer rich in phenolics (particularly hydroxycinnamic acids and flavonoids), protects the top and lower epidermis, which in turn protects the mesophyll (Štambuk et al., 2022).

**FIGURE 1:**
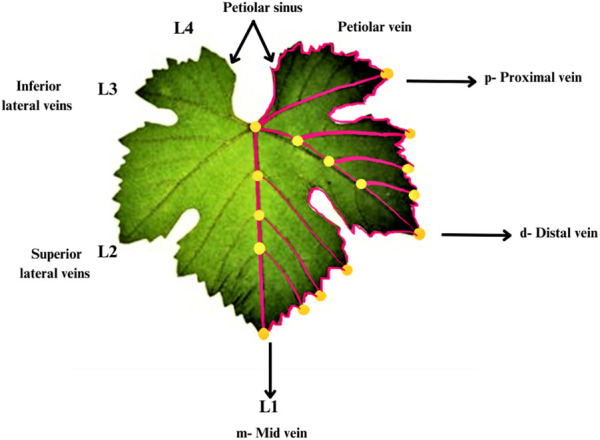
Morphology of grape leaves, Landmarks and pseudo-landmarks plotted of the grape leaf.

There are many factors which determined the form of grape leaves other than serration and lobing. According to the Galet formula and Procrustean method, landmarks of the leaf are plotted in orange dot while pseudo-landmarks are in magenta lines. The regions of the leaf are indicated as’p’- proximal, “d”-distal and, “m” midvein. Midvein as L1, distal/superior as L2, proximal/inferior as L3 and petiolar veins as L4 are provided in the Galet formula and Procrustean methods. According to Galet formula and Procrustean methods, landmarks and pseudo-landmarks are provided which combine and generate vectors, which are styled as black arrows and have their names specified. Each arrow’s base and tip represent the start and finish of a vector along the blade (Figure 1). The direction of the vein vector starting at a similar branch site within the leaf and ending at the tip is indicated by an arrow starting from the vein tip. Gallet’s nomenclature is on the left side of the sheet. L1, L2, L3 and L4 refer to the middle, distal veins (Chitwood, 2021).

## 3 Phytochemical composition of grape leaves of different varieties

The vine-wine industry produces a lot of agricultural waste. The wastes have almost no value and may need to be disposed of at a higher expense. These phenolic compound-containing wastes all serve as a source for high-value goods (Simonetti et al., 2020). Grapevine leaves have been found to contain 132 phenolic chemicals in total (Goufo et al., 2020). The phenolic content of Michele Palier grape leaves, which was 47.76 ± 1.26 g/L, was the highest of all the grape leaf kinds. The amount of non-flavonoids in Maraština was highest at 27.7 ± 0.9 g/L. Narince had the greatest concentration of total flavonoids at 575.25 ± 0.01 g/L. The highest amount of total flavanols, 2.786 ± 0.03 g/L, was found in Merlot. The highest FRAP content found in Merlot was 183.5 ± 2.2 mM. The greatest TEAC ABTS level was found in Yapıncak, which was 524.42 ± 27.51 μmol/g. Narince had the highest antioxidant percentage, which was 88.46% ± 0.03% (Table 1) (Katalinic et al., 2013; Al Juhaimi et al., 2019; Gülcü et al., 2020).

**TABLE 1:** Phytochemical composition of different varieties of grape leaves.

Grape variety	Total phenol (g/L)	Non-flavonoids (g/L)	Total flavonoids (g/L)	Total flavanols (g/L)	DPPH	FRAP (mM)	TEAC ABTS (µmol/g)	Antioxidant activity (%)	References
Maraština	34.5 ± 0.3	27.7 ± 0.9	6.8 ± 0.9	1.1 ± 0.03	52.4 ± 1.2#	106.8 ± 0.9	-	-	Katalinic et al. (2013)
Pošip	46.8 ± 0.4	25.1 ± 0.8	21.7 ± 0.8	2.5460 ± 0.025	71.5 ± 0.7#	140.9 ± 1.6	-	-	Katalinic et al. (2013)
Lasin	32.5 ± 0.3	24.5 ± 0.2	7.9 ± 0.2	1.091 ± 0.031	54.1 ± 0.6#	97.0 ± 0.7	-	-	Katalinic et al. (2013)
Merlot	45.4 ± 0.6	27.5 ± 0.9	17.8 ± 0.9	2.786 ± 0.03	75.4 ± 1.2#	183.5 ± 2.2	-	-	Katalinic et al. (2013)
Syrah	45.8 ± 0.4	22.6 ± 0.4	23.2 ± 0.4	2.202 ± 0.039	69.6 ± 0.5#	162.9 ± 2.2	-	-	Katalinic et al. (2013)
Vranac	37.8 ± 0.7	22.6 ± 0.1	15.3 ± 0.1	2.302 ± 0.015	64.5 ± 1.2#	155.5 ± 2.2	**-**	**-**	Katalinic et al. (2013)
Cabernet Sauvignon	19.49 ± 9.06	-	8.09 ± 2.92	-	17.64 ± 3.93##	-	375.74 ± 82.90	-	Gülcü et al. (2020)
Narince	31.95 ± 1.27	-	575.25 ± 0.01	-	23.65 ± 0.25##	-	398.37 ± 52.96	88.46 ± 0.03	Al Juhaimi et al. (2019), Gülcü et al. (2020)
Trakya İlkeren	33.52 ± 0.63	-	317.74 ± 0.00	-	19.13 ± 0.63##	-	391.36 ± 26.14	84.54 ± 0.01	Al Juhaimi et al. (2019), Gülcü et al. (2020)
Alphonse Lavallee	33.02 ± 0.37	-	11.77 ± 0.29	-	17.62 ± 0.82##	-	393.79 ± 4.27	-	Gülcü et al. (2020)
Hamburg Misketi	32.19 ± 0.13	-	13.10 ± 0.01	-	20.36 ± 1.55##	-	311.59 ± 15.18	-	Gülcü et al. (2020)
Michele Palier	47.76 ± 1.26	-	17.77 ± 0.72	-	18.41 ± 0.42##	-	440.99 ± 106.98	-	Gülcü et al. (2020)
Gamay	31.83 ± 0.13	-	12.86 ± 0.37	-	14.54 ± 1.71##	-	360.09 ± 63.66	-	Gülcü et al. (2020)
Clairette	30.28 ± 0.20	-	11.66 ± 0.42	-	17.16 ± 1.63##	-	332.53 ± 13.14	-	Gülcü et al. (2020)
Yapıncak	46.66 ± 0.08	-	545.39 ± 0.07	-	18.66 ± 2.36##	-	524.42 ± 27.51	86.66 ± 0.03	Al Juhaimi et al. (2019), Gülcü et al. (2020)
Cinsaut	31.44 ± 0.65	-	422.65 ± 0.01	-	20.03 ± 0.28##	-	334.27 ± 5.48	86.58 ± 0.01	Al Juhaimi et al. (2019), Gülcü et al. (2020)
Palieri	11.62 ± 0.08	-	306.06 ± 0.00	-	-	-	-	84.46 ± 0.02	Al Juhaimi et al. (2019)

# = inh%, ## = µmol/g (Katalinic et al., 2013; Al Juhaimi et al., 2019; Gülcü et al., 2020).

It was discovered that total flavonoids (TFC) and phenolic acids were the highest concentration in grape leaves, which were detected in the extract of *V. vinifera*leaf extract (16.75 mg/g total flavonoid content and 6.39 mg/g caffeic acid derivatives). Compared to some grape varieties from Turkey, Croatia, or India, *V. vinifera* leaves from Romania had a greater total flavonoid concentration (Moldovan et al., 2020). Thus, the secondary metabolites of *V. vinifera* are a great source of natural antioxidants that can be used to improve human health.

## 4 Color property of grape leaves

One of the most crucial characteristics for fresh grape leaves is colour (Güler and Candemir, 2014). Based on a global standard suggested by the Commission Internationale d'Eclairage (CIE) and utilised in colour measurements, this colour measurement method was created in 1931. The system was updated in 1976 and given the name CIE L, a, b (Doğan and Cüneyt, 2020). L* stands for lightness in the CIE Lab colour space, which ranges from 0 (black) to 100 (white); a* for redness, which ranges from green (-a*) to red (+a*); and b* for yellowness, which ranges from blue (-b*) to yellow (+b*) (Flamminii et al., 2020). In a 2014 study by Güler and Candemir, the colour characteristics of grape leaves from several grape cultivars were evaluated. By using a Minolta Colorimeter, the samples’ L*, a*, and b* values were determined, and a/b values were computed. Samples’ L* values ranged from 37.92 to 45.0, while their a* and b* values were −8.26 to −3.86 and7.34 to 15.03, respectively (Güler and Candemir, 2014). In some grape cultivars, leaves may have rough surfaces (fluffy, wavy) and different thicknesses. Such differences may result in darker colors on higher or swelling sections (Doğan and Cüneyt, 2020). Fresh green grape leaves were dried by microwave, airand combination of microwave-airto study the effects of thermal treatment on color parameter of grape leaves. In all the drying experiments, the colour criteria that was closest to that of fresh leaves occurred at 500 W-75°C, whereas the color criteria that was furthest from that of fresh leaves occurred at 100°C (Alibas, 2014).

## 5 Extraction techniques for the preparation of grape leaf extract

**FIGURE 2:**
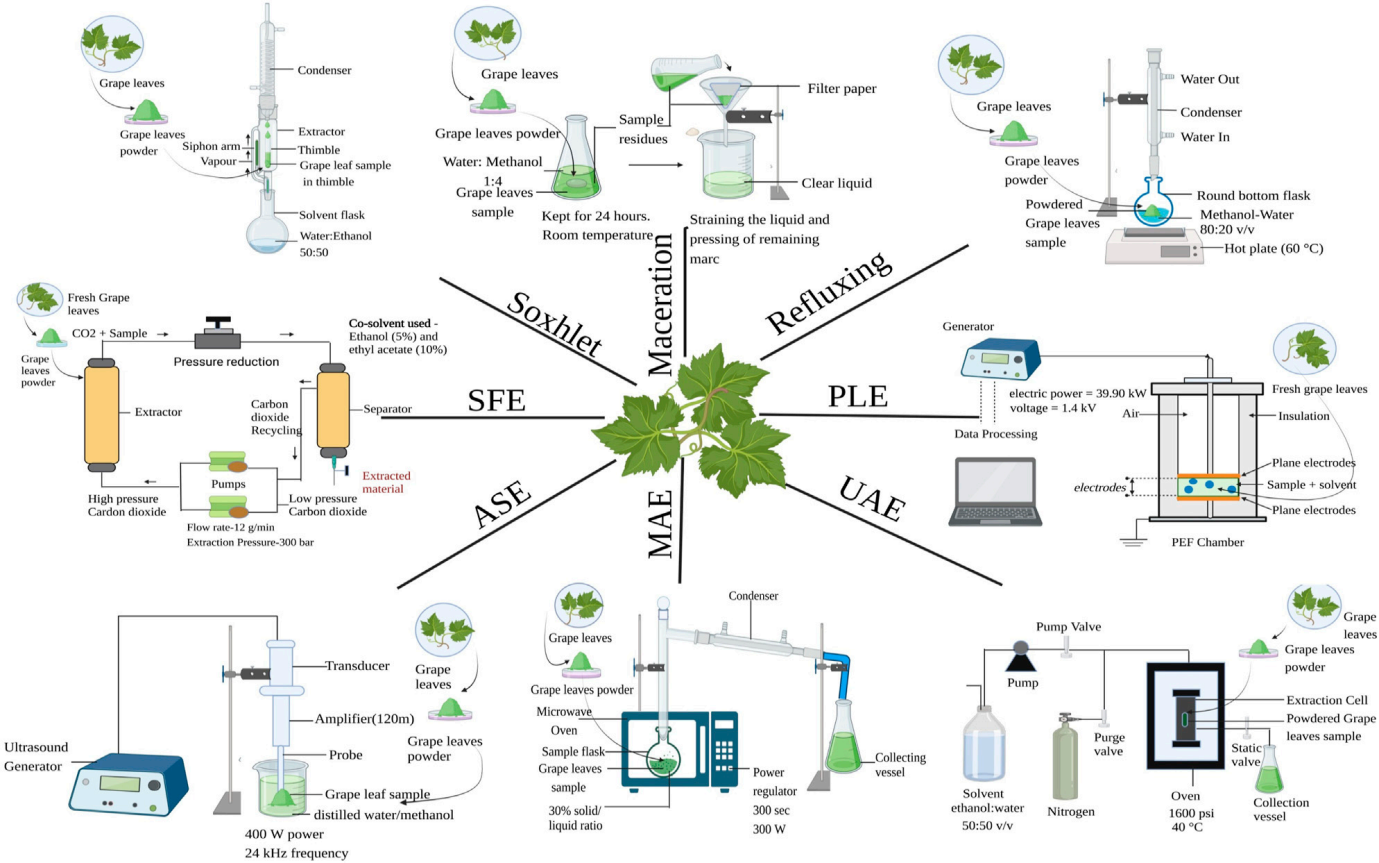
Extraction procedure of grape leaves, MAE = Microwave-assisted extraction, UAE = Ultrasound-assisted extraction, ASE = Accelerated solvent extraction, SFE = Supercritical fluid extraction and PLE = Pulsed Electric Field.

Extraction can be thought of as an intermediate stage between analytical processes and future product production (Picot-Allain et al., 2021). Every extraction method’s primary goal is to optimise the recovery of the desired components from a sample matrix while maintaining the integrity of the relevant molecules and minimising the co-extraction of additional contaminants or undesired chemicals (Garcia-Vaquero et al., 2018). Many biological actions, including as antioxidant, antimicrobial, antispasmodic, anti-inflammatory, anti-allergic, hepatoprotective, and anti-carcinogenic capabilities, can be attributed to the diverse collection of secondary metabolites that are found in plants known as polyphenols (Jovanović et al., 2017). The right extraction technique needs to be taken into account in the first step. The chemical makeup of the component, particle size of the sample, and the presence of interference-causing compounds all have an impact on the choice of extraction method that should be used (BrglezMojzer et al., 2016). The extraction techniques can be categorized into two–conventional and non–conventional extraction techniques as shown in Figure 2.

### 5.1 Conventional techniques

In order to retrieve phenolic chemicals from plant matrix, extraction is a crucial technique (Al et al., 2018a). Maceration, percolation, digestion, decoction, serial exhaustive extraction, infusion and Soxhlet extraction are the conventional techniques used for the extraction ofphenolic compounds (Alara et al., 2021). The majority of these methods rely on the extracting ability of various solventsas well as the use of heat or mixing (Azmir et al., 2013). Traditional extraction techniques are notoptimally consistent since they typically involve using larger amount of extraction solvents and labour-intensive manual processes that are mostly dependent on the researcher (Alara et al., 2021). Liquid-liquid and solid-liquid extraction arethe most popular extraction techniques, in spite of a number of drawbacks. Simplicity, effectiveness, and broad applicability of the conventional methods have led to wide spread acceptance for a long time (BrglezMojzer et al., 2016). The required polyphenol molecules are captured by a variety of solvents, including water, ether, benzene, chloroform, hexane, ethanol, acetonitrile and methanol, when pre-treated plant material obtained by washing, drying, etc., are subjected to it (Suwal and Marciniak, 2018). For extraction of polyphenol from grape leaves following conventional techniques can be used.

#### 5.1.1 Soxhlet extraction

Among the earliest procedures known to be employed for the extraction of polyphenol compounds is Soxhlet extraction, also known as solid-liquid extraction (Suwal and Marciniak, 2018). The solvent is transferred into a distillation flask, and the sample is placed in a thimble-wrapped Soxhlet extractor. In order to make the evaporated solvent useable again, condenser devices are utilised in this extraction process (Tambun et al., 2021). The vapours begin to condense as soon as the heated solvent (ultrapure water:ethanol, 50:50) contacts the condenser and thereafter, the solvent was gathered in the collection vessel. This exhausted extraction procedure was carried out continually for about 6 h until the solvent in the siphon tube turned clear without any extraction material dissolving in it. Then filtration of extract was done and rotary evaporator was used to evaporate the liquid (Labanca et al., 2020). In comparison to more advanced extraction methods like supercritical fluid extraction, this process is not viewed as being environmentally friendly and may worsen the pollution problem (Azwanida, 2015).

#### 5.1.2 Classic solvent extraction

Solvent or liquid–liquid extraction is a two-phase solute distribution wherein two phases of immiscible liquid are placed in proximity with other and the solute is dispersed between them (El-Nadi, 2017). This technique is simple and easy to use. 250 mL of ethanol/water in the ratio 80/20 v/v at 60 °C for 60 min was used as solvent for extraction of 5 g of homogenized grape leaves powder. Whatman No. 1 filter paper was used filter the extract to remove any remaining residue after cooling at ambient temperature for 30 min and 3 × 10 mL of solvent was used to wash the leftover residue (Katalinić et al., 2009).

#### 5.1.3 Reflux extraction

Reflux extraction is a method of solid-liquid extraction in which the solvent is evaporated and condensed for one to 3 hours at a fixed temperature of 60°C–80 °C (Kumar et al., 2023). Compared to percolation or maceration, reflux extraction is more effective as it requires less solvent and extraction time (Zhang et al., 2018). 100 mL of methanol-water solvent in ratio 80–20 v/v was used to extract polyphenols from 40 g of crushed leaves with 0.1 mL/mL of concentrated HCl to prevent its oxidation. A 30-min reflux extraction procedure was carried out at 60 °C. The extracts were then filtered followed by centrifugation at 3,000 rpm for time span of 20 min at 25 °C and later kept in dark at 4 °C (Selka et al., 2019).

#### 5.1.4 Maceration

Maceration is another technique of solid liquid extraction. The inert container is filled with the substance to be extracted, and the solvent is then entirely poured over it (Naviglio et al., 2019). Maceration is a traditional extraction technique that is the simplest and cheapest because it only needs a container for the extraction to take place but it is time consuming. Methanol, ethyl acetate, ethanol and distilled water are typical solvents used in maceration (Tambun et al., 2021). 1,000 mL of water-methanol mixture in the ratio 1:4 was used as solvent for extraction of 150 g of powdered leaves at room temperature by maceration in sealed container for period of 24 h with interval shaking (Borah et al., 2018).

### 5.2 Non-conventional techniques

While there is unquestionably a clear need to develop affordable, safe, efficient, and environmentally innovative extraction methods, it is important that these techniques not only enable clean label status but also ensure increased yields with little impact on the quality of the finished product (Tiwari, 2015). Keeping in consideration the flaws offered by conventional methods, new and advanced green technologies have been developed for better extraction of polyphenolic compounds such as microwave-assisted extraction, ultrasound assisted extraction, pulsed electric field and enzyme-assisted extractions. With the development of new technology, extraction techniques have improved, and are cleaner resulting in products that are less expensive and that do not contain organic solvents (Panja, 2018). Such methods are sometimes referred to as “cold extraction techniques” because temperature used while extracting a molecule is relatively minimal and has little impact on the stability of the isolated compounds (Tiwari, 2015).

#### 5.2.1 Microwave-assisted extraction

The fundamental principle of the MAE technique is the following conversion of microwave energy into thermal energy. Microwaves warm the liquid within the cells of solid samples throughout the MAE. This occurrence raises the pressure inside the cells, which causes the cell wall to degrade. As a result, the sample becomes more porous, allowing the extraction solvent to penetrate the matrix and enhancing extraction efficiency more effectively (Tomaz et al., 2019). The MAE devices can be divided into two categories based on the microwave energy they apply to the sample: Multimode and single-mode system. Multimode systems disperse microwave radiation throughout a space to treat samples uniformly, and single-mode or focused systems apply microwave energy only specific to the sample for a more effective extraction. Focused systems are often employed with open vessels running at atmospheric pressure, whereas multimode systems are applied to closed containers, allowing the simultaneous application of high pressure throughout the extraction process (Gomez et al., 2020). Dipole rotation and ionic conduction—which involves reversing dipoles and dispersing charged ions—are the two ways by which energy is transferred during the microwave heating process. These two systems frequently operate together in various applications. Ions move electrophoretically when an electromagnetic field is applied, and when a solution resists this flow of ions, there is friction that causes the solution to heat up. This process is known as ionic conduction. Dipole rotation entails moving the dipoles around in response to the applied field (Deo et al., 2015).

Several researchers have described MAE as a quick extraction approach that has various benefits over traditional extraction techniques, including high extraction rates, quick extraction periods, and low solvent use (Mustapa et al., 2015). When MAE is used, the extraction time is significantly shortened since microwaves heat the solution directly, whereas with traditional extraction methods, the vessel must first be heated for a certain amount of time before heat is delivered to the solution. Solvent and matrix types, solvent volume, microwave power, exposure time, sample size, moisture content, and temperature are the primary factors affecting MAE performance (Llompar et al., 2019). The rapid rise in temperature of the extraction mixture, which could end the extraction process soon when the solvent boils, is one of the challenges faced by MAE. The extraction yield is decreased when the extraction is stopped too soon because the desired compounds are not properly dispersed into the solvent from the material. In contrast to continuous microwave heating, cooling the extraction mixture with intermittent microwave operation utilising 30 s “on” and 30 s “off” could greatly lengthen the extraction time (Chuyen et al., 2018).

For maximum antioxidant component extraction from grapevine leaves, the ideal microwave conditions were 30% solid/liquid for 300 s at 300 W of microwave power. After being microwaved, samples were centrifuged at 9,000 rpm for time span of 15 min at temperature of 25 °C and filtered using filter paper and supernatants were collected and stored in storage at −18 °C (Hayta and İşçimen, 2021). The primary benefits of MAE over Soxhlet extraction relate to the sharp decrease in solvent consumption (5 V 100 mL) and extraction duration (40S V 6h) (Chaturvedi, 2018).

#### 5.2.2 Ultrasound-assisted extraction (UAE)

Various molecules and biomaterials such as proteins, peptides, fine chemicals, polysaccharides, and bioactive components are extracted from a complex matrix of a plant. Ecological-friendly extraction techniques are required in order to reduce both environmental and health risks which are associated with the use of toxic solvents which have safety risks. Green extraction techniques provide safe, effective-way of extraction of molecules, reduce the use of toxic chemicals/solvents and are affordable which are undoubtedly offers higher extraction yield with improve quality of extract (Tiwari, 2015). The ease of use and affordable equipment make the ultrasound-assisted extraction (UAE) method especially appealing. It is based on the utilisation of ultrasound energy (sound waves that have frequency higher than 20 kHz) to speed up the extraction of compounds from solid samples using a solvent that is chosen based on the characteristics of the solutes that need to be extracted (Carrera et al., 2012).

The water-circulating double-layered mantle enabled cooling and heating systems is used to regulate the temperature during extraction (Both et al., 2014). It is also one among the green technology. Two mechanisms, mechanical mixing effect and ultrasonic cavitation, control the rise in extraction efficiency as well as the decrease in extraction time (Oroian et al., 2020). The primary driving force behind ultrasound‐assisted extraction (UAE), which can create bubbles due to temperature and pressure variations, is acoustic cavitation force. It has a ability to cause a series of compressions and rarefaction in the molecules of current solvent (Medina-Torres et al., 2017). Cell walls and solvent diffusion are broken down with application of high-frequency waves, which perturb the solute-solvent combinations. In order to get a higher efficiency, factors like swelling rate, disruption and particle size post-treatment must be taken into account. Breakdown of particle-particle bond occurs as a result of applied high intensity. When the bonds are broken down, this causes solvent to enter the compounds so deeply and called it as cavitation (Sridhar et al., 2021). Erosion occurs as a result of implosion of cavitations bubbles on the surface of the plant tissues which may potentially be responsible for this degradation. Extraction yield is enhanced by the erosion as area that has been eroded, makes it easier for the solvent to contact it. The release of bioactive components present in the cell membrane of plant is caused by the development of pores during the process of cavitation. This phenomenon is known as sonopration (Kumar et al., 2021). Overall, a number of mechanisms, including fragmentation, erosion, sonocapillary effect, sonoporation, local shear stress, and destruction-detexturization of plant structures, have been found as contributing to ultrasound-assisted extraction (Chemat et al., 2017).

In ultrasound assisted extraction, the sample was weighed at 10% (w/w) solid/liquid ratio into 250 mL beaker then 50 mL of distilled water/methanol was added. Titanium probe (H22D, 22 mm) with 400W power, 24 kHz frequency, and 120 m amplitude was employed with the ultrasound equipment. To prevent the sample from overheating during the ultrasound treatment, a 1000 mL beaker was employed. 100% of the amplitude of ultrasound was used for 10 min. Supernatants were collected, freeze-dried, and kept at 4 °C after being centrifuged at 9,000 rpm for 10 min at 20°C (Hayta and Işçimen, 2018). Despite the widespread use of UAE, the technique has two issues which includes uneven ultrasound energy distribution and a power decrease over time (Ajila et al., 2011).

#### 5.2.3 Accelerated solvent extraction

Accelerated solvent extraction is also known as Pressurized liquid extraction (PLE), it is regarded as advanced extraction technique due to its advantage over conventional extraction techniques. In order to maintain solvent in the liquid state, temperature of the solvent is increased and this allows enhancement of mass transfer rate by decreasing solvent surface tension and viscosity, increases diffusivity. Increased pressure maintains the solvent below its boiling point. The solubility of analytes increases, this allows solvent to penetrate easily into food matrix being extracted (Alvarez-Rivera et al., 2020; Osorio-Tobón, 2020). The benefits of using PLE are higher extraction yield, saves energy since a liquid’s sensible heat is lower than its vaporisation heat. Major solvents employed are water and alcohols in this extraction technique. These solvents are non-toxic and good to the environment. These solvents are inexpensive as a sizable port ion is liquid water in this technique. Equipments used in the extraction process such as extractor and the related setup are simple (Panja, 2018).

The extraction solvent is moved through an extraction cell holding the sample in an accelerated solvent extraction process. The sample cell is heated by direct interaction with the oven. Direct contact between the hot solvent and the sample is used for both static and dynamic extraction. When extraction is finished, compressed nitrogen is used to transport all the solvent from the cell to the vial for analysis. Away from the sample matrix, the filtered extract is gathered and prepared for examination. A broad variety of applications, extractions for sample sizes ranging from 1 to 100 g in minutes, a dramatic reduction in solvent consumption, and handling of both acidic and alkaline matrices are all clear advantages of ASE (Mottaleb and Sarker, 2012). Other advantages include simple automation, quicker sample analysis, higher repeatability, low risk of solvent exposure, and preservation of samples in an oxygen- and light-free environment. Furthermore, by optimising the temperature, pressure, extraction duration, and number of extractions, accelerated extraction systems give the operator better control over the amount of chemicals extracted from the plant (Gomes et al., 2017). Additionally, it has a broad range of applications, from tissues from plants, animals, and environmental toxins, having the capacity to handle both basic and acidic sample (Ahmad et al., 2019). The assays were conducted at 1,600 psi for three rounds of 5 min each. Before being recovered with ethanol and water in ratio 50:50 v/v, the dried leaves were packed into the cell and filled while it was heated to 40°C. It was collected and filtered to extract the mixture (Labanca et al., 2020).

#### 5.2.4 Supercritical fluid extraction

A green extraction method known as supercritical fluid extraction (SFE) has been used extensively to recover high-value components from a variety of materials, at both levels, in laboratories and industries (Chaves et al., 2020). The principle behind supercritical extraction is the transformation of the gas used in supercritical fluid caused by changes in temperature and pressure. A supercritical fluid (SCF) is a fluid whose critical temperature (Tc) and critical pressure (Pc) values are exceeded. Due to its low critical temperature and nonexplosive nature, CO2 is the most widely used SCF. It is also safe, affordable, and has significant benefits for pharmaceutical applications (Fomo et al., 2020). Surface tension is absent at supercritical conditions, or above the critical point, where there is no liquid-gas phase barrier. Fluid contains the characteristics of both a gas and a liquid at the same time and behaves like a single phase. At this point, fluid diffuses like a gas into the solid matrix and dissolves the active components. This is the foundational idea behind supercritical fluid extraction (Panja, 2018). A basic SFE system is made up of the following components: a mobile phase tank for the solvent (typically CO2), a pump for moving and pressurizing the solvent, a cosolvent vessel, a heater for the solvent or supercritical mixture, a pressure vessel for the extraction, a controller to keep the system’s pressure at a high level, and a collection vessel to collect the extract (Chaves et al., 2020).

For extraction of grape leaves Supercritical fluid extractions (SFE) were carried out in a cylindrical 0.5 L lab scale extractor from Applied Separations Inc. (United States) with internal dimensions of 7.3 cm and a height of 12 cm. The supercritical CO2 (SC-CO2) flow rate and extraction pressure were both kept constant at 12 g/min and 300 bar, respectively. Ethanol (E) and ethyl acetate (EA), at concentrations of 5 and 10 weight % relative to the constant SC-CO2 flow rate, were the cosolvents added to the ground particles. The overall yield of the SFE tests utilising crushed (less than 10 mm) and ground (less than 1 mm) biomass ranged from 1.86 to 7.52 wt%, with the best outcomes coming from the extraction of ground particles using SC-CO2 with 10 wt% ethyl acetate at temperature of 80 C (de Melo et al., 2020). Since SFE uses supercritical solvents, which have unique physicochemical properties including density, diffusivity, viscosity, and dielectric constant, it has a number of operational advantages over traditional extraction techniques. Supercritical fluids having a low viscosity and a reasonably high diffusivity, has increased transport capabilities than liquids, the ability to diffuse efficiently through solid surfaces, and the ability to provide faster extraction rates. Other benefits includes the use of solvents that are generally recognised as safe (GRAS), the extraction process’s higher efficiency in terms of increasing yields and cutting down on extraction times, and the potential for direct coupling with analytical chromatographic methods like gas chromatography (GC) or supercritical fluid chromatography (SFC) (Da Silva et al., 2016). Since CO2 exhibits polarity similar to pentane in the supercritical area, it can be used to extract lipophilic compounds. Because CO2 lacks the polarity necessary to remove polar compounds, this is its primary drawback (Yousefi et al., 2019).

#### 5.2.5 Pulsed electric field extraction

Another non-thermal food processing technology with a lot of potential is pulsed electric field (PEF). In the case of PEF treatment, application of the critical electrical potential to cell membranes results in rapid mass transfer, tissue breakdown, and augmentation of tissue permeability (Roselló-Soto et al., 2015).

The electroporation phenomenon of cell membranes, which occurs when a potential difference develops across a membrane, is the foundation of the PEF-assisted approach. The process of molecular orientation known as electroporation occurs when an electric field is present, molecules gravitate towards the membrane by aligning themselves with it. The membrane begins to tear and develop pores as a result of electrocompression. A temporary (reversible) or permanent (irreversible) reduction of membrane permeability can occur from this. For plant cells that range in size from 40 to 200 μm, a critical electric field strength of 1–2 kV/cm determines how much permeability is lost and how much pore development occurs. A high voltage generator, an impulse generator, a Schmitt trigger circuit, a high voltage switch, an oscilloscope, and two plate electrodes (1.4301) make up the setup for pulsed electric field assisted extraction (PEF). A 20 mL mixed glass beaker included plate electrodes with a separation distance d of 0.42 cm and a surface A of 6 cm2. The PEF aided extraction procedure was carried out using fresh red vine leaves at a voltage of U = 1.4 kV, a conductivity of σi = 1.425 mS/cm, and an electric power of Pel = 39.90 kW (Bachtler and Bart, 2018). The PEF continuous extraction can produce larger yields in less time while using less organic solvent and reducing pollutants. Additionally, the entire procedure can be completed at or just a little above ambient temperature. Thus, by avoiding the thermal stimulator, the bioactive compounds won't be damaged. But when the electric field strength is strong enough, there will be substantial concentrations of hydroxyl radicals, which will cause bioactive components to degrade during the PEF extraction (Xi et al., 2021). PEF’s use in the food sector is growing in popularity; it is now regarded as a cutting-edge method for food processing that is suitable for the pre-treatment of edible liquid and semi-solid products. To prevent the dielectric breakdown, it is only applicable to materials with low electrical conductivity and no air bubbles (G et al., 2019).

The microwave-assisted extraction approach has shown the greatest results when compared to other polyphenol extraction techniques. The benefit of microwave extraction is that it may be performed with high efficiency while using the least amount of solvent and least amount of time. Additionally, MAE is superior to conventional extraction techniques in terms of the ease of maintaining extraction containers, improvement of analytical capacities (such as improvement of recovery and reproducibility), and the capacity to simultaneously extract numerous samples (Rajbhar et al., 2015). Table 2 covers different extraction methods for extracting various bioactive components present in grape leaf with bioactive properties. Detail of it is mentioned below:

**TABLE 2:** Extraction Methods for Bioactive Components from Grape Leaves with Bioactive properties.

Extraction methods	Bioactive components extracted	Extraction efficiency	Power requirement efficiency	Bioactive properties	References
Maceration	Gallic acid, 3,4- dihydroxybenzoic acid, (+)-catechin, 1,2-dihydroxybenzene, rutin-trihydrate, quercetin, apigenin-7-glucoside, caffeic acid	19.25w/w%	-	Inhibiting neurological diseases. decreased the expression of inflammatory proteins COX-2 and NF-κB and inhibited the replication of the hepatitis C virus (HCV), which causes chronic cirrhosis	Borai et al. (2017), Borah et al. (2018), Banjanin et al. (2021), Kim and Heo (2022)
Soxhlet extraction	Flavonoid, Gallic and caftaric acid, quercetin-3-*O*-glucoside and quercetin, quercetin-3-*O*-galactoside, quercetin-3-*O*-glucuronide and quercetin-3-*O*-glycoside	30.45%	-	Activation of nuclear erythroid-related factor 2 (Nrf2), resulting to hepatoprotective effect, MAPK and NF-κB signaling pathways were involved in anti-inflammatory processes. reduces the release of inflammatory cytokines, chemokines, adhesion molecules, and cell infiltration, which attenuates the inflammatory response	Brad et al. (2017), Labanca et al. (2020), Bai et al. (2021)
Microwave-assisted extraction (MAE)	Quercetin-3-glucuronide, quercetin-3-glucoside, Kaempferol-3-rutinoside, kaempferol-3-glucoside, and quercetin-3-rutinoside, gallic and caftaric acids, phytosterols	-	300 W	Enhanced expression of nuclear factor erythroid 2-related factor 2 (Nrf2), skin protective effects, anti-inflammatory properties in lipopolysaccharide-stimulated macrophages, anticancer and antioxidant properties, and antimelanogenesis properties in human keratinocytes and melanoma cells through NF-κB and AP-1 pathways	Reis et al. (2015), Djemaa-Landri et al. (2020), Ha et al. (2021), Hayta and İşçimen (2021)
Ultrasound-assisted extraction (UAE)	Resveratrol, Caftaric acid, (+)-Catechin, Benzoic acid, Rutin, Quercetin-3-*O*-galactoside, Quercetin -3-*O*-glucuronide, Quercetin-3-*O*-glycoside, Kaempferol-3-*O*-glucoside	13.81%	%100 amplitude	Anti-obesity, cardioprotective, anticancer, antitumor, antidiabetic, antioxidant, anti-age effects, and glucose metabolism, inhibited CYP2E1, which prevented acetaminophen (APAP)-induced liver damage in mice by controlling oxidative stress by regulating the expression of glutathione-related metabolites and enzymes	Hayta and Işçimen (2018), Hu et al. (2020), Labanca et al. (2020), Paczkowska-Walendowska et al. (2023)
Accelerated Solvent Extraction (ASE)	Caftaric acid, (+)-Catechin, Benzoic acid, Rutin, Quercetin-3-*O*-galactoside, Quercetin -3-*O*-glucuronide, Quercetin-3-*O*-glycoside, Kaempferol-3-*O*-glucoside	6.44%	-	Normalizing the levels of nitric oxide, glutathione, and glutathione peroxidase, as well as reducing oxidative stress and strengthening the antioxidant defense system, protects the liver from oxidative stress-related damage	Babri et al., 2014, Arika et al. (2019), Labanca et al. (2020)
Supercritical fluid extraction	Caffeic acid, catechins	-	-	Antioxidant potential, anti-inflammatory	Arika et al. (2019), Chao et al. (2019), Lefebvre et al. (2021)
Pulsed Electric Field	Flavonoids	-	39.90 kW	Anticholinesterase, pro-cholinergic, anti-inflammatory, antiapoptotic, and antioxidative properties	Borai et al. (2017), Bachtler and Bart (2018), Shiekh et al. (2021)

Gallic acid, 3,4-dihydroxybenzoic acid (+)-catechin, 1,2-dihydroxybenzene, rutin-trihydrate, quercetin, apigenin-7-glucoside, and caffeic acid are the most commonly found bioactive components obtained from grape leaves during the maceration process. It was discovered that the extraction yield of grape leaves was 19.25w/w%. Due to the presence of bioactive components like catechins and their metabolites, which are decomposed into (+)-catechin or (+)-epicatechin and gallic acid which have great bioactivity in reducing inflammation in the body, a variety of bioactive properties have been discovered. These properties may be essential in suppressing neurodegenerative illnesses. In addition to this, by blocking hepatitis C virus (HCV) replication and the production of inflammatory proteins COX-2 and Nuclear factor kappa B (NF-κB) (+)-catechin prevented cirrhosis brought on by a chronic HCV infection, thus helpful in prevention of liver damage (Borai et al., 2017; Borah et al., 2018; Banjanin et al., 2021; Kim and Heo, 2022). The highest extraction yield was observed in Soxhlet (SOX) extraction of about 30.45%. SOX extract of grape leaf tissue allowed the identification of the bioactive components such as flavonoid, gallic and caftaric acid, quercetin-3-*O*-glucoside and quercetin, quercetin-3-*O*-galactoside, quercetin-3-*O*-glucuronide and quercetin-3-*O*-glycoside (Brad et al., 2017; Labanca et al., 2020). One of bioactive components such as gallic acid shows anti-inflammatory property. In order to inhibit the release of inflammatory factors (TNF-α, IL-1β/6), chemokines (CCL-2, ICAM-1, TIMP-1) and other inflammatory mediators (COX-2, nitric oxide), gallic acid attenuates the activation of MAPK and NF-κB/AP-1 signaling pathways (Bai et al., 2021). Detail of Nuclear factor kappa B (NF-κB) and inflammation process is described below (see Section 6.2).

Microwave-assisted extraction (MAE) was used to identify and quantify the bioactive components found in grape leaves, including gallic and caftaric acids, phytosterols, quercetin-3-glucuronide, quercetin-3-glucoside, and kaempferol-3-rutinoside (Djemaa-Landri et al., 2020). Microwave-assisted extraction (MAE) of grape leaves was performed with a power of 300 W (Hayta and Işçimen, 2021). It was discovered that MAE increases yield, shortens extraction times, and uses less solvent (Reis et al., 2015; Hayta and Işçimen, 2021). Numerous studies have revealed that quercetin is associated with melanogenesis and offers pharmacological effects such as anti-inflammatory, anti-atherosclerotic, antioxidant, anticancer, and skin protecting qualities. Quercetin 3-O-β-D-glucuronide (Q-3-G) has been shown to exhibit anti-inflammatory, anticancer, and antioxidant properties in lipopolysaccharide-stimulated macrophages. This has been demonstrated by an increase in Nrf2, one of the key regulators of oxidative stress in cells (Ha et al., 2021). Ultrasound was applied 100% amplitude in extraction of grape leaves through Ultrasound-Assisted Extraction (UAE) (Hayta and Işçimen, 2018). The extraction yield was found to be 13.81%. Resveratrol, Caftaric acid (+)-Catechin, Benzoic acid, Rutin, Quercetin-3-*O*-galactoside, Quercetin -3-*O*-glucuronide, Quercetin-3-*O-*glycoside, Kaempferol-3-*O*-glucoside were identified in grape leaves by the use of UAE. However, gallic and caftaric acid, quercetin-3-*O*-glucoside and quercetin were not present in UAE (Labanca et al., 2020). Numerous biological activities of resveratrol include anti-aging, glucose metabolism, anti-tumor, anticancer, antitumor, antidiabetic, antioxidant, and anti-obesity activities (Paczkowska-Walendowska et al., 2023). These bioactive components possess various bioactivity, one of it has been demonstrated that quercetin-3-O-galactoside, also known as hyperoside, may have therapeutic and preventive benefits for liver disorders. By inhibiting CYP2E1, HPS regulated the glutathione-related metabolites and enzymes, preventing oxidative stress-induced liver damage brought on by Acetaminophen (APAP) (Hu et al., 2020).

The extraction yield was found to be 6.44% with the use of Accelerated solvent extraction (ASE) in grape leaves. Caftaric acid (+)-Catechin, Benzoic acid, Rutin and its derivatives, Quercetin-3-*O*-galactoside, Quercetin -3-*O*-glucuronide, Quercetin-3-*O-*glycoside, Kaempferol-3-*O*-glucoside were found in grape leaves (Labanca et al., 2020). By inhibiting NF-kB activity and prostaglandin synthesis, catechins lower inflammation. By stabilizing the levels of glutathione, nitric oxide, and glutathione peroxidase, quercetin protected the liver from oxidative damage (Arika et al., 2019). The activity of acetylcholinesterase (AChE) is likewise decreased by troxerutin which is derivative of rutin. Acetylcholine (ACh) is a neurotransmitter that is more abundant in the synaptic cleft and facilitates better neurotransmission when AChE activity is reduced (Babri et al., 2014). Although Supercritical fluid extraction is mostly useful for lipid extraction, but it can also be used to extract some polyphenols, including catechins and caffeic acid (Lefebvre et al., 2021). The bioactive activities of these compounds were found to be antioxidant potential, anti-inflammatory (Arika et al., 2019; Chao et al., 2019). Using undried red vine leaves, the Pulsed electric field (PEF) assisted extraction process was executed with an electric power of 39.90 kW and extracted flavonoids a subgroup of ployphenols (Bachtler and Bart, 2018). Numerous studies have demonstrated the potential of pulsed electric field (PEF) with higher energy/intensity or pulses for longer processing times as a non-thermal treatment for increasing yield (Shiekh et al., 2021). The anticholinesterase, pro-cholinergic, anti-inflammatory, antiapoptotic, and antioxidative properties of Vitis leaf polyphenols may contribute to their memory-improving properties and may have a function in the clinical treatment of neurological illnesses (Borai et al., 2017).

## 6 Bioactive properties of grape leaves

**FIGURE 3:**
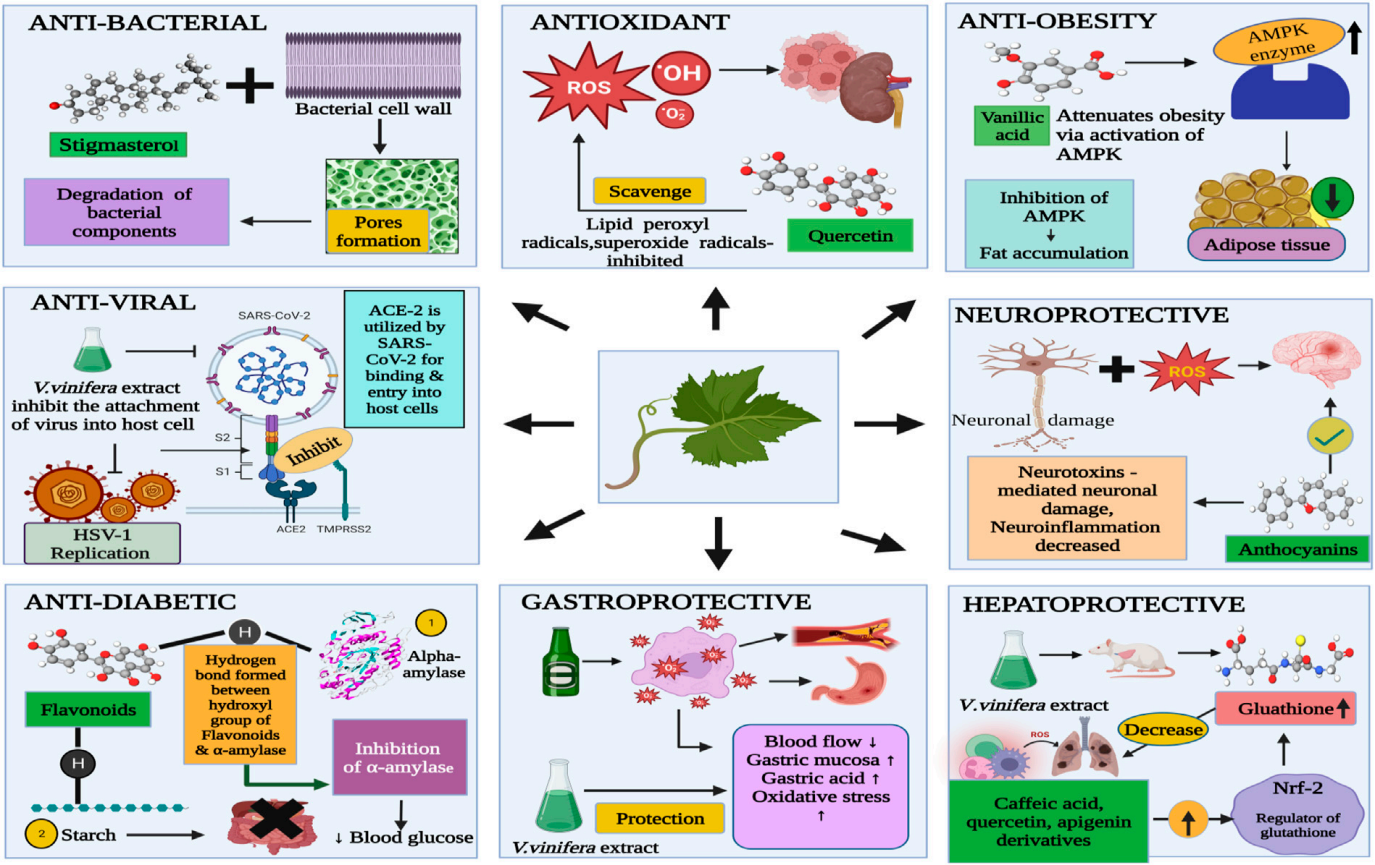
Bioactive properties possessed by active components in grape leaves.

A wide range of benefits from grape leaves and its bioactive components, such as antioxidant, antidiabetic, antibacterial, anti-inflammatory, antiviral, anti-obesity, and hepatoprotective, and gastroprotective activities (Figure 3) could be used in healthcare as products. Bioactive properties of *Vitis vinifera* leaf extract is due to bioactive components present in it which include six hydroxybenzoic acids, namely, 2,5-dihydroxybenzoic acid, protocatechuic acid, p-hydroxybenzoic acid, vanillic acid, gallic acid and syringic acid and six hydroxycinnamic acids, namely, p-coumaric acid, o-coumaric acid, cinnamic acid, ferulic acid, caffeic acid, and sinapic acid (Meng et al., 2021). Table 3 covers experimental studies to represent the bioactive properties of grape leaf.

**TABLE 3:** Bioactive properties of grape leaf.

Bioactive activities	Plant part	Model (cell/animal/humans)	Dosage/concentration	Effects	References
Anti-inflammatory	*Vitis vinifera* water extract (VVWE)	Human keratinocytes HaCaT cells (*in vitro*)	2.5–50 μg/mL	VVWE reduced TNF- or LPS-induced IL-8 production in a concentration-dependent manner. Reduce the NF-B nuclear translocation produced by TNF- by 50% at 50 g/mL	Sangiovanni et al. (2019)
	Hydroalcoholic leaf extract of *Vitis vinifera* (EVV)	Swiss albino mice (*in vivo*)	100 mg/kg, 200 mg/kg, and 400 mg/kg (Doses-1, Soses-2 and Doses-3 respectively)	Doses exhibited maximum anti-inflammatory activity, EVV in addition to indomethacin significantly inhibited acetic acid-induced vascular permeability in mice	Aouey et al. (2016)
	Grape leaves extract (LE)	Human gingival fibroblasts (HGF) cell lines	100, 200, and 300 μg/mL (Doses-1, Soses-2 and Doses-3 respectively)	Significantly impacted the baseline oxidative condition of HGF cells, as well as moderating the proinflammatory response caused by HGF cell LPS exposure	Moldovan et al. (2020)
Anticancer	Ethanolic and aqueous extract of grape leaves	HepG2, MCF-7 cells, HUVEC cells	0.5, 1, 2 mg/mL	The grape leaf extract inhibited the proliferation of MCF-7 breast cancer cells and HepG2 hepatocarcinoma cells	Ferhi et al. (2019)
	*Vitis vinifera* leaf extract	HaCaT cells (*in vitro*)	100 μg/mL	Protective effect against cutaneous damage caused by UV radiation. The quantity of γ-H2AX foci rises. Vitis vinifera L. leaf extract increase system activity or hasten the activation of repair enzymes	Marabini et al. (2020)
Neuroprotective	*Vitis vinifera* leaves polyphenols (VLP) extract	Adult male Wistar rats (*in vivo*)	17 mg/kg body weight	Improved neurobehavioral changes, When AlCl3-intoxicated rats received VLP treatment, serum/brain ACh levels increased significantly, regulating cholinergic function and lowering AChE activity, In AD-induced rats, IL-6 levels increased while serum BDNF levels decreased	Borai et al. (2017)
	Hydroalcoholic extract of *Vitis vinifera* red leaf	Rat astrocyte cell or rat model	145.4 ± 11.4 μM	Lowering oxaliplatin-induced apoptosis, oxidative damage, and cell death. Furthermore, the anti-neuropathic qualities of the same extract were demonstrated when it was examined in a rat model of oxaliplatin-induced neurotoxicity. Reduced astrocyte activation in the spinal cord.Without impairing the anticancer action of oxaliplatin, *vitis vinifera* decreased oxidative damage and reduced pain	Micheli et al. (2018)
	*V. vinifera* leaf extract or *V. vinifera* ethanol (VVE)	Caenorhabditis *Elegans* Model	25, 50, and 100 μg/mL	Protective function against glutamate-mediated oxidative stress-induced cell death in HT22 cells. Increased the expression of genes related to cellular antioxidant enzymes, including CAT, SODs, GSTs, and GPx. Reduces reactive oxygen species buildup and oxidative stress in *C. elegans*. Suppress the expression of hsp-16.2 and increase the expression of the stress-response genes gst-4 and sod-3	Duangjan et al. (2021)
Anti-diabetic	Grapevine leaf extract	Adult male Wistar rats (*in vivo*)	50, 100, or 200 mg kg^−1^	Grapevine leaf extract reduced lipid and protein degradation in diabetic rats’ livers, as demonstrated by decreases in TBARS and carbonyl levels and increases in sulfhydryl levels. SOD activity increased, while CAT activity decreased. STZ rats lost weight, had lower AST levels, and had lower LDL cholesterol	dos Santos Lacerda et al. (2014)
Anti-obesity	Methanolic and aqueous extract of *Vitis vinifera* (VVME) and (VVAE)	Wistar male rats	100 mg/kg, 200 mg/kg and 400 mg/kg (Doses-1, Soses-2 and Doses-3 respectively)	Lowered the glucose level, Reduced the disruption of the endothelium lining, atherogenic index, and lipid profile, and attenuated the high SGOT and SGPT levels. The aortic wall thickening was also improved	Devi and Singh (2017)
	Vitis vinifera L. leaf extract (VLE)	Mice		Inhibit the pancreatic lipase activity. Mice administered a low-density lipoprotein (LDL) diet in addition to intraperitoneal injection (VLE) showed a significant reduction in body weight, tissue fat accumulation, cholesterol, and triglyceride levels over time. Showed higher levels of fibroblast growth factor 15 and lower levels of neuropeptide-Y (NPY) in the serum and hypothalamus. Regulate energy metabolism and body weight gain, VLE controls both the NPY-mediated pathway and the bile acid-FGF15 pathway	Meng et al. (2021)
Anti-viral	Grape leaf extract	Vero cell line, HCT-116, A549, MCF7, H9c2, HepG2 and Hacat cell lines	0.625, 1.25, 2.5, 5, 10, 50, 100, and 500 μg/mL	Both HSV-1 and SARS-CoV-2 replication are inhibited in the early stages of infection by directly inhibiting the proteins abundant on the viral surface. Leaf extract inhibits viral envelope binding to the cell membrane. After SARS-CoV-2 infection, V. vinifera extract significantly reduced S expression	Zannella et al. (2021)
Hepatoprotective	Ethanol along with GLEt	Adult male albino Wistar	(25 mg/kg)	The activities of the liver marker enzyme in serum were considerably reduced by ethanol and GLEt to a level close to normal. GLEt was much more effective at a dosage of Dose3 as per body weight than it was at dose1 and dose2. Furthermore, lipid peroxidation and addition were considerably decreased by GLEt, and both enzymatic and non-enzymatic antioxidants were significantly restored level in the kidney and liver of rats given alcohol. According to data, GLEt improves antioxidant status and reduces lipid peroxidation to have its protective effect	Pari and Suresh (2008)
			(50 mg/kg)		
			(100 mg/kg)		
			Dose1, Dose2 and Dose 3 respectively		
	Grape leaves	Male albino rats	11.89g/day	Helpful in enhancing liver functions and might shield rats against acute liver disease damage brought on by (CCL4)	Negam et al. (2020)
			12.31g/day		
			Dose1 and dose 2 respectively		

### 6.1 Antibacterial property

Various medicinal plants contain an unsaturated phytosterol known as stigmasterol or stigmasterin (Mohan et al., 2022). Stigmasterol has been considered as bactericidal property in grape leaf extract. Both gram-positive and gram-negative bacteria are strongly inhibited by Stigmasterol, which means that Stigmasterol is potential as an antimicrobial agent (Khumaidah et al., 2019). Bacterial components are degraded by stigmasterol by means of two ways-surface interaction of stigmasterol with bacteria and forming pores in the bacterial cell wall. Other reasons of showing antimicrobial effect of grape leaves are due to inactivation of microbial adhesions by tannins present in grape leaves, capacity of it to improve the morphology of microorganisms (Sharma et al., 2021).

### 6.2 Anti-inflammatory property

Anti-inflammatory effect of grape leaves is due to components present in it, which include kaempferol, resveratrol, quinic acid and quercetin (Ins et al., 2021). During process of inflammation, reactive oxygen species (ROS) and nitrogen species are involved which causes cellular damage, moreover release of free radicals causes oxidative stress. There are certain transcription factors such as Tumor necrosis factor (TNF-α) and interleukins (ILs) which are released and resulting in inflammation, these mediators are further directly and indirectly induced by ROS (Reactive oxygen species) and RNS (reactive Nitrogen species). More free radicals are produced and this continued during the whole inflammatory process (Direito et al., 2021). Anti-inflammatory activity of *Vitis vinifera* has been showed by acting on NF- κB pathway and decrease of IL-8 (Interleukin- 8) in Tumor necrosis factor alpha (TNF- α), which is cytokine. Fibroblast-like synoviocytes (FLS) cells and macrophages produce Interleukin (IL) such as IL-6, causes release of chemokines by stimulating endothelial cells and also activates lymphocytes like B and T cells. Vascular endothelial growth factor (VEGF) production, which is responsible for pannus formation, is actively facilitated by IL-6. Macrophages produce IL-1β in the inflamed synovium (Ali et al., 2023). Various pathways are activated by these inflammatory cytokines to induce inflammation such as NF-κB(Nuclear factor kappa B). During inflammatory process, IL- 8 is a chemokine released by keratinocytes (Sangiovanni et al., 2019).

Nuclear factor kappa B (NF- κB) regulate important physiological processes such as inflammation, immunity, cell growth and death of cell and targets the defense mechanism of the body by regulating expressions of certain genes in the process including cytokines and pro-inflammatory cytokines (Zinatizadeh et al., 2021), represents plays an important role in a variety of conditions of skin-inflammation including psoriasis which refers to an inflammatory dermatosis, indicated by high levels of NF- κB (Goldminz et al., 2013). *Vitis vinifera* has capability to inhibit the NF- κB (Nuclear factor kappa B) pathway through the impairment of translocation from the cytoplasm into the nucleus in human gastric epithelial cells (Vinceković et al., 2019). The highest dose of Leaf extract causes reduction in levels of interleukins such as IL-6 by 60% and IL-8 by 40%, whereas IL-1β concentration was reduced to the basal level by the extract (Moldovan et al., 2020).

### 6.3 Antioxidant activity

Antioxidant plays an important role in delaying, inhibiting or preventing oxidation of oxidizable materials by scavenging free radicals and thus oxidative stress is diminished. Powerful antioxidants have been considered *in vitro* to be more vigorous than Vitamin C and E, carotenoids, some of derivatives of hydroxycinnamic acid such as caffeic and chlorogenic acid and flavonoids such as quercetin and its derivatives, which possesses antioxidant activity in vine leaves. Vine leaves extract inhibited peroxidation of linoleic acid due to strong activity possess by compounds present in leaves extract (Bajomo et al., 2022). Caffeic acid is found in many of plant-based foods sources which involves in biosynthetic pathway as intermediate, moreover phenolic acids and precursor molecule of lignin are also produced by caffeic acid. Due to resonance stabilization of structure of caffeic acid, it have a high antioxidant potential than other compounds such as hydroxycinnamates such as ferulic acid (Chao et al., 2019). One of bioactive component of vine extract such as Quercetin is responsible for the increase of the levels of enzymatic and non-enzymatic antioxidants, thus reducing oxidative stress and improving the antioxidant defense mechanism (Babri et al., 2014). Quercetin has ability to scavenge some of radicals, namely, superoxide radicals, hydroxyl radicals, and lipid peroxyl radicals through its antioxidant activities. Quercetin supplements protects the liver from damage caused by oxidative stress by normalizing the levels of nitric oxide, glutathione and glutathione peroxidase as shown by previous studies in mice (Babri et al., 2014).

### 6.4 Neuroprotective activity

In elders, Alzheimer disease is the most common cause of decline in memory and neurocognitive function, which involves formation of amyloid-b (Ab) plaques, neurofibrillary tangles (NFTs), cholinergic dysfunction, and oxidative pathogenic factors (Babri et al., 2014).

In humans and animals, a decline in motor and cognitive could be more susceptible to the effects caused by oxidative stress and inflammation (Zhang et al., 2013). *Vitis vinifera* leaves contains a wide range of polyphenols such as anthocyanins, flavonoids and also organic acids, namely, malic acid, oxalic acid and tartaric acid is found. Polyphenols present in vine leaves interacts with various pathways including neuronal and glial signalling. In addition to, vine leaves also decrease ROS production, neuronal damage caused by neurotoxins and neuroinflammation, resulting in improvement of neurological health (Borai et al., 2017).

### 6.5 Anti-obesity property

The antiobesity effect of many phenolic acids has been reported. Vanillic acid reduces obesity by the activation of the AMPKpathway (AMP-activated protein kinase) (Mohan et al., 2022), AMPK (AMP-activated protein kinase) acts as energy homeostasis, as an enzyme it is involved in the energy metabolism of in liver, muscle and fat tissue, inhibition of AMPK is one of the most important cause of accumulation of liver fat. The enzyme ACC, i.e., Acetyl-CoA carboxylase was phosphorylated by the activation of AMPK. Malonyl-CoA synthesis was enabled by the ACC enzyme, which potentially inhibits fatty acid oxidation in mitochondria (Jung et al., 2018).

Mature, lipid-containing adipocytes are converted from preadipocytes in a process of proliferation is called adipogenesis (Jakab et al., 2021). Adipocyte differentiation was accelerated by reactive oxygen species (ROS) production, whereas differentiation was diminished in the presence of antioxidants. The hydroxybenzoic structure of syringic acid, containing methoxyl groups in positions 3 and 5 and a hydroxyl group in 4, was made a potent antioxidant (John and Arockiasamy, 2020). The ability of syringic acid to inhibit adipogenesis and promote lipolysis in adipocytes, possibly through a reactive oxygen species (ROS)-mediated mechanism, had demonstrated its anti-obesity effect (Meng et al., 2021).

### 6.6 Anti-viral effect

V.vinifera leaf extract showed a strong antiviral activity against two important human pathogens, i.e., HSV-1 and severe acute respiratory syndrome coronavirus 2 (SARS-CoV-2) (Zannella et al., 2021). The host angiotensin-converting enzyme 2 (ACE2) was utilized by SARS-CoV-2, like SARS-CoV-1, for binding and entry into host cells (Zhao et al., 2020).

V.vinifera leaf extract inhibit the attachment of both of the viruses, namely, HSV-1 and SARS- CoV-2 into the host cell. *V. vinifera* hinders some of interacting site inside the viral glycoprotein deputed to the fusion, i.e., HSV-1 glycoprotein b and SARS-CoV-2 spike protein, process of formation of infection, binding of viral envelope with cell membrane are prevented. Much of the viral gene expression was blocked by the extract and the replication of HSV-1 was inhibited, almost having at a concentration of 10 μg/mL. The literature demonstrating the leaf extract had antiviral action and results were also supported. Leaf extract could be used as a prophylactic treatment for herpetic infections (Zannella et al., 2021).

### 6.7 Anti-diabetic activity

The anti-diabetic activity of *V. vinifera* leaves was suggested to be contributed to by condensed tannins and flavonoids (Negam et al., 2020). The degree of inhibition of alpha amylase enzyme was investigated by estimating antidiabetic activity of leaf extracts of *Vitis vinifera* (Dar et al., 2022).

The inhibition mechanism of alpha amylase is still unclear. However, starch and nitrous acid which are derived from the oral cavity can be interacted with flavonoids present in food in the stomach, this interaction is occurred before transportation to the intestine. Two mechanisms of action are mainly seen, (a) flavonoids showing mechanism of action by inhibiting activity of α-amylase activity, and (b) flavonoids interacts with starch through hydrophobic interactions and resulting in the formation of starch-flavonoids complex which leads to suppression of starch digestion. The inhibition of tannins is correlated to the number of hydroxyl groups in the B cycles of alpha-amylase. Hydrogen bonds are formed between the OH group (hydroxyl group) of these compounds and the residues of the active site of the alpha-amylase, causing the alpha-amylase to be inhibited. Diabetes risk can be reduced by flavonoids-rich food sources through the modulation of glucose uptake and insulin secretion (Mohammed et al., 2020).

It had been shown by a report that various potential diabetes inhibitors from plants which can inhibit α-amylase and α-glucosidase belong to flavonoid group (Al et al., 2018b).

### 6.8 Gastroprotective effect

The phenolic compounds of hydroxymethanolic extracts of two varieties of grape leaves, i.e., "Nefza-I″ and “Marsaoui” can protect the damage of gastric mucosa which is caused by the ethanol alcohol by scavenging free radicals as per shown in the report. The anti-ulcerogenic effect is attributed to decrease blood flow and enhanced oxidative stress, changes in the mucosal barrier caused by severe choroid plexus lesions, and the well-known ruptured blood vessels that result from ethanol consumption. Gastric acid secretion was inhibited, stimulation of mucus and bicarbonates were seen and gastroprotective mechanism of action represent by increases blood flow on gastric mucosal. Pre-treatment of rats with grape leaves extract at the levels of 100 mg/kg and 200 mg/kg were given, it has been found that this showed higher effects than the pre-treatment of rats with omeprazole. In this sense, the lesion formation was prevented and hydroxymethanolic extract of grape leaves extract promoted better health of gastric mucosa as a gastroprotective effect even more than the drugs used for gastric ulcers (Saadaoui et al., 2020).

### 6.9 Hepatoprotective effect

Ascorbate and glutathione or Glutathione peroxidase scavenge reactive oxygen species directly or indirectly by the principal antioxidant, particularly reduced glutathione (GSH), known as glutathione (Sahoo et al., 2017). During metabolism and immune response of the body, levels of ROS increase, therefore in order to maintain optimum levels of glutathione, upstream regulators, such as nuclear factor E2-related factor 2 (Nrf 2) are essential to increase or decrease synthesis and recycling of the molecules (Teskey et al., 2018).

The activity of Glutathione reductase (GR) is increased in rats treated with ethanol alcohol, moreover, recycling of reduced glutathione is increased which further adds up to the increase of activity, while radical scavenging activity is decreased by the decrease in Glutathione peroxidase (GPx). Grape leaf extract could normalise the oxidative stress that occurs in the liver by above mentioned markers. Activation of nuclear erythroid-related factor 2 (Nrf2) by secondary metabolites present in the extract (e.g., apigenin derivative, epicatechin, quercetin derivatives, caffeic acid, rosmarinic acid derivative, and isorhamnetin derivatives) may be involved. partly attributed to the antioxidant effects observed for hepatoprotective effect (Amen et al., 2020).

## 7 Food and non-food applications of grape leaves

Grape leaf has potential application in developing various value-added products. In food applications, grape leaf extract is formulated in bakery products; beverages and it provide health benefits. In non-food applications, grape leaf has been used and act as preservative, it has been used in cosmetics and played a role in anti-hyperpigmatation. Few researchers have shown grape leaves utilization as traditional and processed foods, other researchers have shown the use of grape leaves in non-food applications (Table 4) (Dogan et al., 2015; Hefnawy et al., 2016; Cosme et al., 2017; Lin et al., 2017; Jridi et al., 2019; Capone et al., 2021; Kaur et al., 2023).

**TABLE 4:** Application and utilisation of grape leaves.

Product	Formulation	Findings	References
Biscuits	Grape leaf extract, sugar, fat, sodium and ammonium bicarbonate, sodium chloride, wheat flour	After baking, GLE had preserved 51.0% of its activity. Ash levels in biscuits enhanced with GLE were comparable to those in the control sample. The breaking force was lowered by GLE. All samples peroxide values (PV) increased after 15 days of storage, but the GLE-enriched sample’s PV spiked suddenly from 0.034 to 0.374, indicating that it was ineffective at preventing the oxidation of lipids	Hefnawy et al. (2016)
Sarma	Grape vine leaves, rice, minced meat, vegetables, and seasonings	-	Dogan et al. (2015), Cosme et al. (2017)
Pistachios Calissons	Vine leaf powder, pistachio paste and meringue (sugar, egg white, lemon and rose water)	Increased antiradical activity of the calissons, reduced its peroxide value from 2.6 ± 0.1meq/kg to 1.5 ± 0.1meq/kg, increased fat retention	Jridi et al. (2019)
Shiraz Wine	Shiraz fruit, grapevine leaves, grape rachis or peduncles	Certain compounds such as 1,1,6-trimethyl-1,2-dihydronapthalene (TDN), 2-methylpropanol, β-damascenone, 2- and 3-methylbutanol, hexanol and (Z)-3-hexen-1-ol were found in highest amount in wine made with grapevine leaves, carotenoid content in wine made with grapevine leaves was found to be enhanced by 8 times as compared to wine made with grape pulp	Capone et al. (2021)
Cosmetics	Red vine leaf extract (RVLE) with an IC50 of 3.84 mg/mL	Various substances to treat hyperpigmentation caused by tyrosinase enzymes. Red vine leaf extract (RVLE) was used as an inhibitor against tyrosine activity, with an IC50 of 3.84 mg/mL. RVLE’s lowering of tyrosine activity makes it safe for cosmetic use	Lin et al. (2017)
	Dried leaves of *V. vinifera*Jingzaojing (100 g) 1 L of 3% acetic acid - 30% ethanol	The mice treated with 3% acetic acid-30% ethanol had longer maximal running times, greater distances, and longer run times until exhaustion. Red grape leaf extract also increased the expression of enzymes involved in fatty acid oxidation and mitochondrial biogenesis	Lee et al. (2022)
Enhance Endurance Capacity	Red grape leaf extract (RGLE)	It was found that RGLE improved mice’s endurance capacity by stimulating lipid catabolism in the muscle. The 0.5% RGLE group showed a 27% increase in activity compared to the control group. Swimming times of mice fed RGLE increased in a dose-dependent manner, and endurance capacities were higher by 11.4% and 34.3% compared to control mice. After swimming activity, RGLE had a 21% greater plasma NEFA (non-esterified fatty acids) concertation	Minegishi et al. (2011)
Tablet	Red vine leaf extract (RVLE)	Antistax (tablet 360 mg) with a dosage of 700–1000 mg per day improve clinical symptoms related to Chronic venous insufficiency (CVI). Results showed that edema was decrease with 86 mL in volume. It improves venous flows and oxidative stress was reduced by 28%	Belcaro (2015), Belcaro et al. (2017), Azhdari et al. (2020)
Preseravtive	Grape leaf extract (GLE)	(i) Grape leaf and stem extracts have been micro-capsulated and used as food preservatives. Grape byproducts, such as leaves have strong antioxidant properties due to their high phenolic content. Microencapsulated grape byproduct extracts can increase food viability and protect it from degradation. It can lower ochratoxins levels and increase shelf life and act as bio-preservative	Badr et al. (2021)
		(ii) The study investigated the effect of grape leaf extract (GLE) on lettuce polyphenol oxidase (PPO) inhibition and phenolic component loss during storage. Heat-treated GLE had a stronger inhibitory effect than freshly extracted GLE. Heat-treated GLE noncompetitively inhibited lettuce PPO, but GLE stopped lightness loss in lettuce stored for storage. GLE’s widespread occurrence, low cost, and non-toxicity make it a potential natural PPO inhibitor. Further feasibility studies are needed to maximize its effectiveness in the food industry	Altunkaya (2014)

### 7.1 Food applications of grape leaves

After the grapes are processed, grape leaves are collected as a significant waste in the agriculture industry (Zannella et al., 2021). In traditional medicine, grapevine leaves are already used to treat bleeding, inflammation, diarrhoea, and diabetes-related hepatic problems because they are relatively common plant material and contain a variety of phenolic chemicals and antioxidants (Maia et al., 2019).

#### 7.1.1 Bakery products

Walid and Rania (2023) state that fats are necessary for dough production since they give the desired flavour. Not only can nutritious values vary as a result of lipid oxidation that occurs in biscuits, but it also produces organoleptic alterations. Consumers find biscuits to be undesirable due to a bad taste, a lack of desired texture, and an unpleasant odour. According to Walid and Rania (2023) and Mildner-Szkudlarz et al. (2022) lipid oxidation in biscuits is caused by polyunsaturated fatty acids included in the shortening used to prepare the dough. Lipids that are preserved in their natural state give optimal texture and dimensional properties while extending the shelf life of biscuits (Gebreselassie and Clifford, 2016).

A study conducted by Hefnawy and El-Shourbagy (Ferhi et al., 2019) carried out an experiment in order to determine the antioxidant activity of some plant extracts, such as Grape leaf extract (GLE), and to determine the stability of lipids included in cookies. Biscuits were prepared enriched with grape leaf extract at 1% (w/w) level. To make biscuits, sugar and fat were first whipped, then extracts, dough water containing sodium and ammonium bicarbonate, sodium chloride, and wheat flour were added. The dough was sheeted, baked, cooled and stored. The biscuits with GLE were the least moist. The ash content in biscuits is another crucial factor. According to the current investigation, the ash concentration of biscuits containing GLE was comparable to that of the control. Moisture and ash content of the biscuits was 3.99% ± 0.09% and 1.28 ± 0.01 respectively while that of the control sample was 4.18 ± 0.08 and 1.26 ± 0.01. When GLE was added, the amount of force needed to break the biscuits decreased. All sample’s peroxide values (PV) increased after 15 days of storage, but the GLE-enriched sample’s PV spiked suddenly from 0.034 to 0.374, indicating that it was ineffective at preventing the oxidation of lipids. Regarding color, crumb colour, texture, and mouth feel, the biscuits made using GLE received favourable reviews.

Greek cuisine has long utilised grape leaves in form of the traditional dish “Sarma”. Sarma, which translates to “wrapped” in Turkish, refers to leaves that are either raw or more frequently quickly blanched, or held in salt brine, and then rolled around a stuffing of rice, bulgur, or minced meat, along with other potential vegetables and seasoning plants particularly onion, before being gently cooked by stewing or boiling in a pot and typically eaten warm with meat or cold without the addition of meat (Marabini et al., 2020). It is one of the most well-liked savouries from the south-eastern European and Middle Eastern cuisines. It is prepared as a side dish or as a main component for a meat supper. The leaves do, in fact, carry a hint of the flavour and aroma of grape (Cosme et al., 2017).

Jridi et al. (2019) utilised Vine leaves powder (VLP) in pistachio calissons. A local pastry shop made pistachio calissons using the usual ingredients: pistachio paste and meringue. The three formulations that were developed included the control formulation referred as F0, F1 enhanced with 3% VLP, and F2 which was enhanced with 5% VLP. The findings of the texture analysis of calissons indicated hardness dramatically increased for F2 from 7.3 ± 1.2 N to 9.5 ± 0.2 N. The final product’s elasticity and cohesiveness were not significantly affected by VLP enrichment. The antiradical activity of the calissons increased because of VLP enrichment. Several samples’ peroxide values grew over the course of storage. The pistachio calisson’s oxidation, however, was significantly reduced by the addition of VLP from 2.6 ± 0.1meq/kg to 1.5 ± 0.1meq/kg,. One factor influencing its appeal was the color of the pistachio calisson. The 3% enriched pistachio calisson retained its characteristically greenish color due to the chlorophyll-rich grape leaves powder. For formulations F1 and F2, the odour and taste ratings were also favourable. It was permitted to add 3% PFV to pistachio calisson.

#### 7.1.2 Beverage

In a study by Capone et al. (2021), grapevine leaves were used in the production of Shiraz wine, and the effects of the leaves on the flavor and volatile composition of the wine were examined. Grapevine leaves were used as MOG (material other than grapes) in this investigation. Shiraz fruit was first used to make wine before 500g of grapevine leaves (or around 1% w/v) were added to each batch along with other components such grape rachis or peduncles. Wine yeast was added to each juice and must in a subsequent step, and fermentation was carried out at a temperature of 15°C–19 °C. Wines were filtered after fermentation was finished and kept at 15 °C for analysis. C6 alcohols at higher amounts, including hexanol and (Z)-3-hexen-1-ol, were studied in wine that had grapevine leaves added. According to a different study, grape leaves had higher levels of C6 alcohols than grapes because they had more enzymatic activity. Carotenoid concentration in wine made with the inclusion of grapevine leaves was enhanced eight times higher in comparison to grape pulp, and C13 norisoprenoids, such as 1,1,6-trimethyl-1,2-dihydronapthalene (TDN), which is responsible for flavour, were more abundant in grape leaves. A number of other substances, including 2-methylpropanol, β-damascenone, 2- and 3-methylbutanol, were present in considerable concentrations. According to the sensory study performed by a panel of nine assessors trained by AWRI, wine created with grapevine leaves had positive fragrance characteristics like “vanilla,""floral,""confection,""sweet,” and “overall fruit".

### 7.2 Non-food/applications of grape leaves

Grape leaf extract has been formulated in various non-food products (discussed below) in order to represent its benefits. In non-food applications, grape leaf has been used in order to preserve the food by increasing shelf life of product, played an essential role in cosmetics.

#### 7.2.1 Cosmetics

In the cosmetics market of Asia, a wide variety of substances are uses for the production of products related to skin care in order to treat hyperpigmentation which occur as a result of enzyme named tyrosinase which causes skin pigmentation which catalyse the reaction in which L-tyrosine is oxidized to DOPA, then to dopachrome (Lin et al., 2017). At the end of the whole reaction process, melanin is produced, responsible for pigmentation of skin. According to Lin et al. (2017), in order to inhibit the activity of tyrosine, red vine leaf extract (RVLE) was used as an inhibitor against tyrosine activity. 3.84 mg/*mVL* was determined as the IC50 for the RVLE solution. Tyrosine’s activity was proven to be lowered by RVLE, which has been demonstrated to be safe for usage in cosmetics.

In a study done by Lee et al. (2022) on mice, *Vitis vinifera* leaf and shoot extracts were given to the animals as supplements. *V. vinifera* Jingzaojing dried leaves and stems (100 g) were treated with 1 L of 3% acetic acid-30% ethanol for 3 h at 90 C. The extracted solution was centrifuged for 10 minutes at 13,000 rpm, and the supernatant was filtered, lyophilized, and powdered. Comparing the mice in the JLSE-supplemented group to the mice in the control group, it was clear that the JLSE-supplemented group had considerably longer maximal running times, greater running distances, and longer run times until exhaustion. Another study found that red grape leaf extract boosts the expression of certain enzymes involved in fatty acid oxidation and mitochondrial biogenesis.

Grapes are one of the top 20 raw fruits in terms of consumption, according to the Code for Federal Regulations (21CFR101, subpart C), and they are governed as foods by the Food and Drug Administration (FDA). The following are evaluated as being safe in this report: 24 Ingredients produced from *Vitis vinifera* (grape) for use in cosmetic formulations includes: *Vitis vinifera* (grape) leaf extract, *Vitis vinifera* (grape) leaf oil, *Vitis vinifera* (grape) leaf/skin/seed extract, *Vitis vinifera* (grape) leaf water, *Vitis vinifera* (grape) leaf wax (Fiume et al., 2014). *Vitis vinifera* derived ingredients are used in the formulations of cosmetics products (Table 5) (Devi and Singh, 2017).

**TABLE 5:** 24 *Vitis vinifera* (grape)-derived ingredients for use in cosmetic formulations.

24 *Vitis vinifera* (grape)-derived ingredients			Reference
*vitis vinifera* (grape)	*vitis vinifera* (grape) leaf extract	*vitis vinifera* (grape) seed powder	Fiume et al. (2014)
*vitis vinifera* (grape) bud extract	*vitis vinifera* (grape) leaf oil	*vitis vinifera* (grape) shoot extract	Fiume et al. (2014)
*vitis vinifera* (grape) flower extract	*vitis vinifera* (grape) leaf/seed/skin extract	*vitis vinifera* (grape) skin extract	Fiume et al. (2014)
*vitis vinifera* (grape) fruit extract	*vitis vinifera* (grape) leaf water	*vitis vinifera* (grape) skin powder	Fiume et al. (2014)
*vitis vinifera* (grape) fruit powder	*vitis vinifera* (grape) leaf wax	*vitis vinifera* (grape) vine extract	Fiume et al. (2014)
*vitis vinifera* (grape) fruit water	*vitis vinifera* (grape) root extract	*vitis vinifera* (grape) vine sap	Fiume et al. (2014)
*vitis vinifera* (grape) juice	*vitis vinifera* (grape) seed	Hydrolyzed grape fruit	Fiume et al. (2014)
*vitis vinifera* (grape) juice extract	*vitis vinifera* (grape) seed extract	Hydrolzsed grape skin	Fiume et al. (2014)

#### 7.2.2 Red grape leaf extract (RGLE) to enhance endurance capacity

According to study by Minegishi et al. (2011), the Mediterranean region has traditionally used red grape leaf extract (RGLE) as a natural medicine. According to a study, mice’s endurance was noticeably improved by red grape leaf extract (RGLE). In the Mediterranean region, venous insufficiency is treated with red grape leaves Multiple polyphenolic substances, including stilbenes, anthocyanins, flavanols, flavonols, flavons, and ellagitannins, are prevalent in red grape leaf extract (RGLE). The study was conducted on endurance capacity in mice. Three experimental groups of male BALB/c mice were created; the control group, the 0.2% (w/w), and the 0.5% RGLE group. All three groups had comparable swimming times and body weights. During the 10-week trial period, swimming times were assessed once a week to assess endurance capacity. Blood analysis of mice was taken. At week 10, the 0.5% RGLE group’s endurance capacity was much longer than the control groups, indicating that red grape leaf extract (RGLE) improved endurance capacity in a dose-related way while simultaneously stimulating lipid catabolism in the muscle. In the muscle, RGLE increased the expression of genes involved in fatty acid oxidation and boosted the activity of fatty acid oxidising enzymes by 27% with the 0.5% RGLE group. After exercise, plasma lactate levels in the 0.5% RGLE group were considerably lower than those in the control group. When compared to control mice, the endurance capacities of mice fed 0.2% and 0.5% RGLE were higher by 11.4% and 34.3%, respectively.

#### 7.2.3 Tablets

Azhdari et al. (2020) state that CVI is a condition caused by inadequate venous return from the lower limbs and is linked to an increase in venous hypertension. The flavonoids in red vine leaf extract shown impacts on venular endothelium and healing of endothelial cell lesions, as well as effects on inflammatory indicators and reaction process of inflammation in the pathogenesis of CVI. This is the mechanism underlying the effect of red vine leaf extract in CVI. Nearly all studies where red vine leaf extract reduced oxidative stress and edema showed improvements in symptoms associated to CVI. In an experiment, Belcaro (2015) compared the effects of two distinct plant extracts on chronic venous insufficiency (CVI). Red grape leaf extract (GLE) is the active component in Antistax (tablet 360 mg), a registered trademark of Boheringer-Ingelheim, Germany. CVI with moderate or severe clinical symptoms was quantified with antistax. 200 subjects were pre-selected, of which 183 were included (with 17 being dropped due to certain personal issues). Out of 166 patients, 55 subjects took the GLE/Antistax medication and finished the 8-week study. Results indicated that issues with leg fatigue and leg aches that are related to a working lifestyle have decreased. Even after prolonged standing or sitting, the walls of the individuals’ leg veins were maintained, ensuring a healthy circulation. As a result, it aids in keeping a regular venous flow and reducing edema symptoms. Two capsules of Antistax were advised to be taken daily for at least 6 weeks.

Another study conducted by Belcaro et al. (2017) compared products used to control symptoms of CVI. Antistax (360 mg of red grape leaf extract) was used as an active ingredient with dosage of 700–1000 mg per day. Results for edema were seen well with a decrease of 86 mL in volume. It was regarded safe-self treatments in several countries. Oxidative stress was also reduced with Antistax of about 28%. It was found that Reactive oxygen species (ROS) levels in the blood was significantly decreased and findings suggest that patient might benefit from Antistax regarding complications associated with T2DM such as wound healing.

#### 7.2.4 Preservatives

Grape leaf and stem extracts were micro-capsulated and used as food preservatives. Epicatechin, quercetin, and rutin were all found in higher concentrations in the leaf extract than in the other. Previous research has shown that phenolic acids have a variety of biological effects, including the ability to kill bacteria. Phenolic acids present in grape leaf showed antifungal properties, including apigenin, luteolin, catechin, chlorogenic acid, and epicatechin. Due to their high phenolic component content, grape byproducts (leaves and stem) have strong antioxidant properties. Grape by-product (including leaves) extracts microencapsulated could increase food viability and the bioactive ingredients could shield it from degradation, used in fruit-based food models such juice and ready-to-eat fruit demonstrated the capacity to lower ochratoxins levels and increase shelf life. Grape byproduct extracts could be used as an innovative method of food bio-preservation (Badr et al., 2021).

Altunkaya (2014) conducted a study to determine how grape leaf extract (GLE) affected the inhibition of lettuce polyphenol oxidase (PPO) and the loss of phenolic components in freshly cut lettuce during storage. The addition of GLE that had been heated at 100C for 15 min had a stronger inhibiting effect on the lettuce PPO activity than the freshly extracted GLE did. Heat-treated GLE was discovered to noncompetitively inhibit lettuce PPO. In lettuce that was being kept track of for storage, the addition of GLE stopped lightness loss. According to the findings of the current investigation, grape leaf extract (GLE) significantly inhibited PPO and stopped the enzymatic browning of freshly cut lettuce. As a result of its widespread occurrence, low cost, and non-toxicity, GLE has the potential to be used as a natural inhibitor of PPO. However, additional feasibility studies are required to maximise effectiveness of grape leaf extract as an anti-browning agent in its use in the food industry.

## 8 Toxicity and recommendations

The extent to which a material can endanger people or animals is known as its toxicity. Acute and sub-acute toxicity are the two different forms of toxicity (Štambuk et al., 2022). Acute toxicology experiments identify the LD50, or median lethal dose, as dosage level at which half of the animals’ death take place. The greatest concentration at which a tested substance does not exhibit any negative effects, also known as the NOEL (No Effect Level), is established in sub-acute toxicity tests (Abbas Momtazi-Boroje et al., 2017).

In a study, ten male Wistar albino rats, each weighing between 180 and 200 g were examined for acute toxicity. Five rats per group were used to separate the animals into control and test groups. Orally administered to the test group of rats was a powder made from grape leaves at a dosage of 5,000 mg/kg body weight. The same volume of distilled water was given to control rats. After extract administration, animals were closely monitored for the following 24 h and 14 d. The rats were monitored for toxicity, morphological changes, and death. The investigated extract’s median lethal dose (LD50) exceeded 5,000 mg/kg body weight, as evidenced by the absence of any obvious toxic effects, aberrant behaviours, or mortality following a single oral dosage of 5,000 mg/kg body weight of *V. vinifera* leaf extract. All animals were alive for 14 days. Based on this the effective dose of (500 mg/kg) was chosen (Sayed et al., 2016).

## 9 Future perspectives and conclusion

The importance of leaves as a medicinal property in the food industry is highlighted by the growing interest in using various types of leaves that were considered waste. This review focuses on the phytochemical composition of the various grape leaf varieties that are grown throughout the world and their potential for bioactivity in the food business. The significant effects of grape leaves as an antioxidant, anti-inflammatory, anti-obesity, anti-viral, and hepatoprotective agent are demonstrated by pharmacological research. Each bioactive component acts on the body differently and through different pathways to ensure its functionality. Due to the high incidence of non-communicable diseases including cancer, cirrhosis, arthritis, etc., dietary habits and consumption of people have evolved to emphasise eating a balanced diet and preventing these diseases. Even though leaves have nutritional benefits, the current food market prioritises producing products at low costs that are affordable to the majority of the population. This is made possible by using inexpensive food materials like grape leaves, which are typically discarded and considered waste. Consumer demand for low-cost items rises as a result of the use of agricultural waste, which ultimately boosts economic growth and the food market. Grape leaves have a range of health advantages, including antioxidant, anti-diabetic, anti-bacterial, anti-obesity, anti-inflammatory, antiviral, hepatoprotective, and gastroprotective effects. Grape leaves are advantageous for use in the management of healthcare because they are nutrient-rich and include bioactive substances such as flavonoids, phenolic acids, antioxidants, and phenols. In addition to being used as a meal and in their raw state in many countries as traditionally, grape leaves have been utilised in baking. In the future, a variety of food items made from grape leaves could be developed that is useful in addressing problems related to nutrition. For the best utilisation of the bioactive components present in grape leaves, the use of grape-leaf-based value-added food products should be supported.
