# Supplementary figures and images for: Valorization of grape (Vitis vinifera) leaves for bioactive compounds: novel green extraction technologies and food-pharma applications
Source: Front Chem. 2023 Dec 12;11:1290619. doi: 10.3389/fchem.2023.1290619 (PMC10754528; doi:10.3389/fchem.2023.1290619)

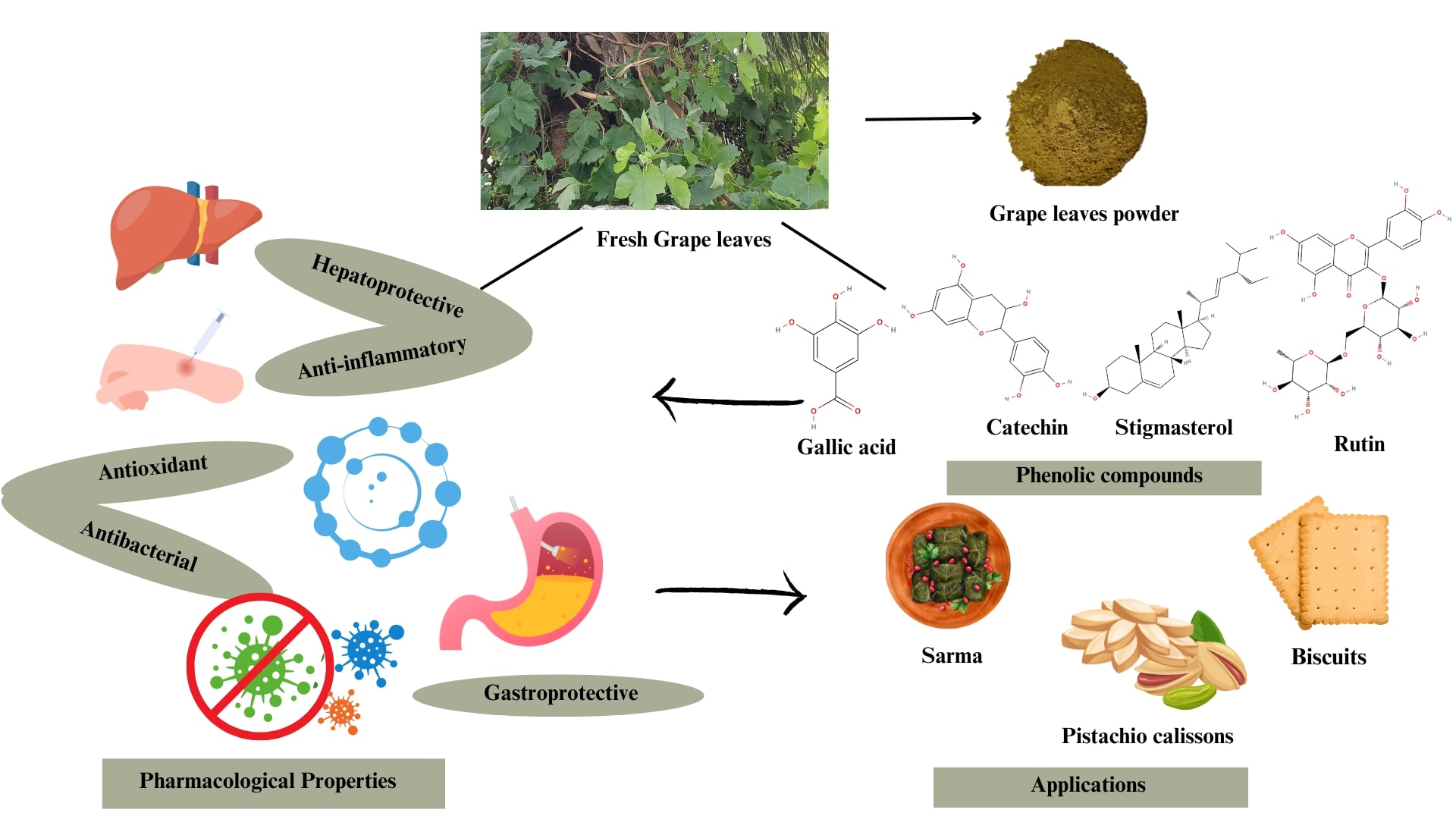

Supplement: Supplementary file 1 [file Image1.JPEG]
